# Differences in HIV testing and receipt of results between adolescent and non-adolescent women in Uganda

**DOI:** 10.1186/s12981-019-0233-3

**Published:** 2019-08-12

**Authors:** Stephen Ediru, Robert Wamala, Betty Kwagala

**Affiliations:** 1 Department of Population Studies, School of Statistics and Planning, Makerere University, P.O BOX 7062, Kampala, Uganda; 2 Department of Sociology and Social Administration, Kyambogo University, P.O BOX 1, Kyambogo, Kampala, Uganda; 3 School of Statistics and Planning, Makerere University, P.O BOX 7062, Kampala, Uganda

**Keywords:** HIV testing, Receipt, Result, Adolescents, Non-adolescents, Decomposition

## Abstract

Despite notable increase in HIV testing among Uganda’s women from 25% in 2006 to 71% in 2011, HIV testing among adolescent women remains very low at 45.5%. This study assesses differences in HIV testing and receipt of results (HTR) between adolescent and non-adolescent women in Uganda. The differences were decomposed into components attributed to variation in characteristics and variation in effects of characteristics in the two groups. The assessment was based on data sourced from 2011 Uganda Demographic Health Survey. Statistical analysis was done using a Non-linear Oaxaca’ Blinder Multivariate Decomposition of the logistic regression. In the results, the difference in HIV testing and receipt of result between adolescent and non-adolescent women was significantly (P < 0.05) attributed to both variation in characteristics (57.2%) and variation in the effects of characteristics/coefficients (42.8%). In particular, the gap in HTR was mainly attributed to variation in characteristics such as ever had sex (34.7%) and ever given birth (31.6%) and variation in effects of characteristics such as education level (− 68.8%) and marital status (− 12.6%). Based on the findings of the study, government and other development partners need to scale up HIV testing programs targeting adolescents through tackling stigma, increasing on community outreach services and expanding adolescent friendly HIV services center.

## Background

Globally, Acquired Immunodeficiency Syndrome (HIV/AIDS) pandemic remains a major challenge to public health [19]. By the end of 2012, an estimated 35.3 million people were living with HIV worldwide [20]. Out of the 35.3 million people, 2.1 million were adolescents, with female accounting for 56% [20]. This pattern of HIV infection is not surprising considering the early adolescents’ initiation into sexual activity. For instance, in Uganda, 60% of young women had initiated sexual activity by age 18 years in 2011 [17].

HIV testing and receipt of results is the gateway to HIV/AIDS prevention, treatment and care programs [19]. HIV testing has been recommended as a routine health care service for young people [3]. However, evidence shows that 53.3% of people living with HIV globally do not know their HIV status and yet timely access to treatment and related prevention services require knowledge of one’s HIV status [5]. By 2011, only about 1/3 of young people knew their HIV status [11]. In sub-Saharan Africa, almost 90% of people who tested positive for HIV went on to access Antiretroviral Therapy (ART) and evidence indicates that 76% of people on ART have achieved viral suppression [6]. Research evidence has demonstrated that fear of HIV testing is associated with limited consent, little privacy and breaches of confidentiality [7]. While adolescent women generally fear test result, married women fear reactions from their husbands in case the HIV test result is positive [2].

Despite tremendous increase in HIV testing and receipt of results among Uganda’s women from 25% in 2006 to 71% in 2011, HIV testing among adolescent women has remained very low at 45.5% [16]. Research evidence reveals that acceptance of HIV testing among women is associated with greater number of antenatal care visits, residing in the urban areas, having comprehensive knowledge on HIV and positive partner’s reaction for HIV positive result [9]. Twenty-five percent of Uganda’s population is comprised of adolescents [17]. Being young and female in Africa tends to increase female adolescents’ sexual risks and vulnerabilities to HIV [8]. Increasing access to and uptake of HIV testing is critical to reduce the incidence of HIV and to improve access to treatment and support for sero-positive people. People who are aware of being HIV-positive are less likely to engage in sexual risk behavior and people who receive antiretroviral treatment (ART) and adhere to it are less likely to infect others [12]. Moreover, stigma is likely to decline because as more people become aware of their sero-status, it becomes normal for them to voluntarily disclose their HIV sero-status [15].

Uganda is one of the Sub-Saharan countries with the highest HIV/AIDS prevalence (7.3%). Women in Uganda bear the biggest burden of HIV prevalence (8.3%) compared to men (6.1%) [18]. Low HIV testing and receipt of results has been associated with increased risky sexual behaviors, infections and delayed access to early treatment, care and support among women [4]. Recent studies on the determinants of HIV testing among women in Sub-Saharan Africa, Uganda inclusive are routinely assessed statistically using methodologies in which age is only included as a covariate [1, 10–12, 21]. Certainly, these approaches provide insight into the relative contributions of different age groups [13]. However, these approaches are limited in terms of partitioning differences in HIV testing into a component attributed to difference in composition of women by characteristics and a component attributed to difference in behavior as a result of difference in characteristics between adolescent and non-adolescent women. In particular, the extent to which difference in HIV testing can be attributed to compositional differences between adolescent and non-adolescent women remains unanswered. Likewise, the extent to which the difference is attributed to the behavioral differences between adolescent and non-adolescent women is yet to be established.

## Methodology

The study utilized data from 2011 Uganda Demographic Health Survey (UDHS). As part of the requirements, authors submitted a proposal to DHS Program/ICF International and permission was granted to download and use data for the study. DHS authorized data access, and the data was used only for the purpose of the current study. This data is from a nationally representative sample of households obtained at two- stage cluster sampling (UBOS and ICF International, 2012). The first stage involved the selection of cluster sample; this was followed by selection of households. Stratification of urban and rural areas was taken into account. The UDHS for 2011 was based on four data collection tools but for this investigation only women’s questionnaire was used. In the 2011 UDHS, women 15–49 were asked if they knew a place where they could go to be tested and further if they had ever undergone an HIV test and received the results of the test. The data was collected from ten regions of Uganda. Women were grouped adolescent aged 15–19 (n = 2048) and non-adolescent aged 20–49 (n = 6648). Data was weighted to ensure representativeness of the sampled data.

## Variables

The dependent variable was HIV testing and receipt of results. The variable had two outcomes: tested and received result or not tested/tested but did not receive result. The independent variables include; Place of residence (urban, rural), Education level (no education, primary, post-primary), region (Kampala, Central 1, Central 2, East Central, Eastern, North, Karamoja, West-Nile, Western, Southwest), Religion (Catholics, Protestant, Muslim, Pentecostal, Other), Wealth index (Poorest, Poorer, Middle, Richer, Richest), marital status (Never in union, married, widowed/separated), ever given birth (yes, no), ever had sex (yes, no), comprehensive knowledge on HIV (have comprehensive, did not have comprehensive knowledge) and HIV stigma (have HIV stigmatizing attitudes, did not have HIV stigmatizing attitudes). HIV stigma was generated from four variables namely willingness to take care of a family member with HIV/AIDS, willingness to buy fresh vegetables from HIV positive vender; allowing a HIV positive teacher who is not sick to continue teaching and would not want to keep secret that a family member has HIV/AIDS. On the other hand, Comprehensive knowledge was generated from variables such as; knowing that consistent use of condom during sexual intercourse and having just one uninfected faithful partner can reduce chance of getting AIDS virus; rejecting misconceptions namely; AIDS can be transmitted by mosquito bite and a person can get infected by sharing food with an infected person.

## Statistical analysis

Statistical analysis was undertaken using STATA 12.0 at three levels: First, a descriptive summary indicating differentials in women’s characteristics was performed using frequency distribution. Secondly, difference in HIV testing and receipt of results between adolescent and non-adolescent women distributed according to their characteristics was performed using frequency distribution showing percentage difference. Thirdly, Multivariate decomposition model which was used to portion differences in HIV testing and receipt of result between adolescent and non-adolescent women into components attributable to variation in characteristics and variation in effects of characteristics between the two groups. All associations were deemed statistically significant at a cut-off P-value of 0.05.

## Results

This section of the paper presents results of the study including percentage distribution of women by characteristics, percentage distribution of women by HIV testing and receipt of results, differences in HIV testing and receipt of results between adolescent and non-adolescent women by characteristics and decomposition of differences in HIV testing and receipt of results.

### Difference in composition of women by characteristics

The characteristics of women assessed in the study were; level of education, place of residence, region, religion, wealth index, marital status, ever given birth, ever had sex, comprehensive knowledge on HIV and HIV stigma. Table 1 presents distribution of adolescent (15–19) and non-adolescent (20–49) women by these characteristics.

**Table 1:** Distribution of women by characteristics

Characteristics	Adolescents (n = 2048)	Non-adolescents (n = 6626)	Difference
Residence			
Urban	19.3	19.9	0.6
Rural	80.7	80.1	− 0.6
Education			
No education	2.9	16.0	13.1
Primary	64.8	57.7	− 7.1
Post-primary	32.3	26.3	− 6.0
Region			
Kampala	9.3	9.8	0.5
Central 1	11.2	10.9	− 0.3
Central 2	9.7	10.6	0.9
East central	9.9	10.1	0.2
Eastern	15.5	14.3	− 1.2
North	8.8	8.4	− 0.4
Karamoja	3.2	3.4	0.2
West-Nile	6.2	5.6	− 0.6
Western	14.0	14.1	0.1
Southwest	12.2	12.8	0.6
Religion			
Catholic	40.6	40.6	0.0
Protestant	29.2	30.2	1.0
Muslim	13.4	12.9	− 0.5
Pentecostal	13.7	13.2	− 0.5
Other	3.1	3.1	0.0
Wealth index			
Poorest	15.4	18.2	2.8
Poorer	16.9	18.6	1.7
Middle	18.0	18.7	0.7
Richer	23.5	18.8	− 4.7
Richest	26.2	25.7	− 0.5
Marital status			
Never in union	77.3	8.1	− 69.2
Married/living together	19.9	75.6	55.7
Widowed/separated	2.8	16.3	13.5
Ever given birth			
No	81.9	8.2	− 73.7
Yes	18.1	91.8	73.7
Have comprehensive knowledge			
No	64.4	61.6	− 2.8
Yes	35.6	38.4	2.8
Ever had sex			
No	54.9	2.4	− 52.5
Yes	45.1	97.6	52.5
Have HIV stigmatizing attitude			
No	80.9	76.8	− 4.1
Yes	19.1	23.2	4.1

The assessment is based on weighted data

From Table 1, notable difference in distribution of women by their characteristics was observed in the following variables; marital status, ever given birth, ever had sex, HIV stigma, comprehensive knowledge, wealth index and education level.

The findings in Table 1 reveal that majority of women in this study were rural residents (80.7% of the adolescent women and 80.1% of the non-adolescent women). In terms of education attainment, more women had primary level of education across all the two groups (64.8% of the adolescents and 57.7% of the non-adolescents). However, about 16% of non-adolescent women had no education compared to only 2.9% of the adolescent women. Findings on regional distribution indicate that in both groups, more women were from eastern region (15.5% of the adolescent and 14.3% of the non-adolescents) and western region (14.0% of the adolescents and 14.1% of the non-adolescents).

Results in Table 1 further show that 40.6% of women in both groups were Catholics. Similarly, most of women were in the richest (26.2% of adolescents and 25.7% of the non-adolescents) and richer wealth quintile (23.5% adolescents and 18.8% non-adolescents). Almost all (97.6%) non-adolescent women had ever had sex compared to only 45.1% of their adolescent counterpart. While majority of adolescents were never in union (77.3%), more of the non-adolescents were married (75.6%). Similarly 91.8% of the non-adolescent women had given birth compared only 18.1% of the adolescent women. In both groups, more women did not have comprehensive knowledge on HIV (64.4% of the adolescents and 61.6% of the non-adolescent women). Less stigmatizing attitude was reported in both groups (23.2% among non-adolescents and 19.1% among adolescent women).

### HIV testing and receipt of results

As earlier on noted, HIV testing and receipt of results was assessed based on binary outcome which included; tested and received results or never tested/tested and never received results.

**Fig. 1:**
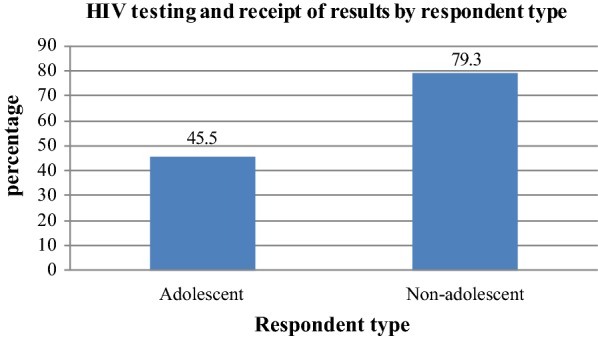
Distribution of women by HIV testing behavior

In the results according to Fig. 1, only 45.5% of adolescent women tested and received HIV results compared to 79.3% of non-adolescent women who tested and received results. This result leaves a very big difference in HIV testing and receipt of results between adolescents and non-adolescent women in Uganda (33.8%) implying that there was low uptake of HIV testing among female adolescents compared to non-adolescent women.

### Differentials in HIV testing and receipt of results

Table 2 presents differences in HIV testing and receipt of result between adolescent and non-adolescent women distributed by their characteristics.

**Table 2:** Differences in HIV testing and receipt of results between adolescent and non-adolescent women

Characteristics	Adolescents (2048)		Non-adolescent (6626)		Differences in HIV testing and receipt of result
	Tested and received result	Never tested/tested but never received result	Tested and received result	Never tested/tested but never received result	
Residence	P = 0.000	χ = 18.638	P = 0.000	χ = 127.923	
Urban	51.8	48.2	87.3	12.4	35.5
Rural	44.0	56.0	77.4	22.6	33.4
Education	P = 0.000	χ = 56.515	P = 0.000	χ = 253.432	
No education	26.7	73.3	67.1	32.9	40.4
Primary	42.2	57.8	79.1	20.9	36.9
Post-primary	53.8	46.2	87.5	12.5	33.7
Region	P = 0.000	χ = 52.961	P = 0.000	χ = 249.999	
Kampala	49.5	50.5	86.3	13.7	36.8
Central 1	47.3	52.7	81.2	18.8	33.9
Central 2	44.0	56.0	79.0	21.0	35.0
East central	37.1	63.9	70.3	29.7	33.2
Eastern	45.6	44.4	78.9	21.1	33.3
North	59.3	40.7	88.6	11.4	29.3
Karamoja	41.4	58.6	68.2	31.8	26.8
West-Nile	44.2	55.8	74.2	25.8	30.0
Western	48.2	51.8	79.3	20.7	31.1
Southwest	36.9	64.1	79.3	20.7	42.4
Religion	P = 0.845	χ = 1.394	P = 0.021	χ = 11.587	
Catholic	49.8	50.2	79.5	20.5	29.7
Protestant	41.8	58.2	78.4	21.6	36.6
Muslim	46.1	53.9	79.2	20.8	
Pentecostal	41.7	58.3	80.4	19.6	38.7
Other	38.5	61.5	83.0	17.0	44.5
Wealth index	P = 0.137	χ = 6.980	P = 0.000	χ = 145.165	
Poorest	48.5	51.5	76.5	23.5	28.0
Poorer	42.8	57.2	75.5	24.5	32.7
Middle	44.3	55.3	76.1	23.9	31.8
Richer	40.9	51.1	79.1	20.9	38.2
Richest	50.3	49.7	86.7	13.3	36.4
Marital status	P = 0.000	χ = 220.149	P = 0.134	4.020	
Never in union	36.3	63.7	75.1	24.9	38.8
Married	75.6	24.4	80.2	19.8	4.6
Widowed	84.1	15.9	77.6	22.4	− 6.5
Ever given birth	P = 0.000	χ = 249.607	P = 0.000	χ = 21.377	
No	37.4	62.6	70.5	29.5	33.1
Yes	82.0	18.0	80.1	19.9	− 1.9
Comprehensive knowledge	P = 0.000	χ = 14.549	P = 0.000	χ = 94.603	
No	43.3	56.7	76.2	23.8	32.9
Yes	49.4	50.6	84.4	15.6	35.0
Ever had sex	P = 0.000	χ = 329.890	P = 0.000	55.528	
No	27.1	72.9	54.0	46.0	26.9
Yes	67.8	32.2	80.0	20.0	12.2
HIV stigmatizing attitude	P = 0.000	χ = 41.355	P = 0.000	χ = 53.300	
Yes	42.7	57.3	77.5	22.5	34.8
No	57.3	42.7	85.5	14.5	28.2

Assessment is based on weighted data

Results presented in Table 2 above indicate a significant relationship between residence and HIV testing and receipt of result in all the two groups (P = 0.000). HIV testing and receipt of result was found to be high among non-adolescent women in both urban (87.3%) and rural (77.4%) areas compared to female adolescents. Highest level of education attained also indicated a significant association with HIV testing and receipt of result in both adolescent and non-adolescent women (P = 0.00). In all the two groups, HIV testing and receipt of result was high among women with post-primary level of education while non HIV testing was significantly high among women with no level of education attained (73.3% in adolescents and 32.9% in non-adolescents).

With regard to region, HIV testing and receipt of result among female adolescents was high in Kampala (49.5%), West (48.2%) and Central 1 (47.3%) while among non-adolescent women HIV testing and receipt of result was high in North (88.6%), Kampala (86.3%) and Central 1 (81.2%). HIV testing and receipt of result also increased for women in the richest wealth quintile (50.3% in adolescents and 86.7% in non-adolescents). However, there was no significate relationship between HIV testing and wealth quintile among female adolescent women (P = 0.137).

HIV testing only indicated significant association with marital status among female adolescent (P = 0.000) and no association among non-adolescent women (P = 0.134). In all the two groups, HIV testing was high among women who are married. Similarly, increase in HIV testing has been associated with women who have ever given birth (82% for adolescents and 80.1% for non-adolescents). Increase in HIV testing uptake was also found to be high among and with no stigmatizing attitudes towards people living with HIV/AIDS (57.3% for adolescent women and 85.5% for adolescent women).

### Decomposition of differences in HIV testing and receipt of results

A multivariate decomposition logistic regression model was used to decompose differences in HIV testing and receipt of results (HTR) between adolescents and non-adolescents women attributed to variation in their characteristics/endowments (E) and variation in the effects of the predictors/coefficients (C). Tables 3 and 4 present decomposition results of differences in HIV testing and receipt of results between adolescent and non- adolescent women.

**Table 3:** Summary of decomposition of HTR

Components	Coef.	p-value	Percent (%)
E	0.1932	0.000	57.21
C	0.1449	0.000	42.79
R	0.3387	0.000	100

Overall decomposition results of HIV Testing and Receipt of results (HTR); n = 8674; variations are attributed to differences due to endowments (E) and coefficients (C); R is the total variation

**Table 4:** Decomposition of HIV testing and receipt of results (“000”)

Variables	Due to difference in characteristics (E)			Due to difference in coefficients (C)		
	Coefficient	P-value	percent	Coefficient	P-value	percent
Education						
No education	1.000			1.000		
Primary	− 8.394	*0.000*	− 2.48	− 141.880	*0.016*	− 41.88
Post-primary	− 13.733	*0.000*	− 4.05	− 90.469	*0.005*	− 26.70
Place of residence						
Urban	1.000			1.000		
Rural	0.568	*0.003*	0.17	− 49.461	0.302	− 14.60
Religion						
Catholics	1.000			1.000		
Protestants	− 0.184	0.335	− 0.05	9.818	0.401	1.91
Muslim	0.010	0.934	0.01	6.458	0.324	1.91
Pentecostal	− 0.050	0.746	− 0.01	8.607	0.242	2.54
Others	0.001	0.405	0.00	3.270	0.284	0.97
Region						
Kampala	1.000			1.000		
Central 1	− 0.182	0.104	− 0.05	− 0.759	0.939	− 0.22
Central 2	0.335	0.371	0.10	− 3.096	0.700	− 0.91
East central	− 0.089	0.298	− 0.03	− 1.924	0.811	− 0.57
Eastern	− 0.089	0.072	− 0.26	− 1.158	0.927	− 0.34
North	− 0.993	*0.000*	− 0.29	− 5.155	0.512	− 1.52
Karamoja	0.050	0.614	0.01	− 6.021	0.078	− 1.78
West-Nile	− 0.283	0.221	− 0.08	− 4.775	0.342	− 1.41
Western	0.024	0.075	0.01	− 7.560	0.529	− 2.23
Southwest	0.748	*0.004*	0.22	0.874	0.689	0.26
Wealth index						
Poorest	1.000			1.000		
Poorer	− 0.552	0.166	− 0.16	0.365	0.689	0.077
Middle	− 0.233	0.232	− 0.07	− 0.650	0.527	− 1.92
Richer	0.162	0.896	0.08	0.708	0.959	0.21
Richest	− 0.001	0.168	− 0.07	− 3.204	0.868	− 0.95
Marital status						
Never in union	1.000			1.000		
Married/living together	− 4.819	0.822	− 1.4	− 33.948	*0.008*	− 10.02
Widowed/separated	− 5.886	0.822	− 1.7	− 7.017	*0.017*	− 2.07
Ever given birth						
Yes	1.000			1.000		
No	106.900	*0.000*	31.55	− 1.574	0.153	− 4.65
Ever had sex						
Yes	1.000			1.000		
No	116.500	0.000	34.72	− 8.119	0.779	− 2.40
Have comprehensive knowledge						
No	1.000			1.000		
Yes	1.369	*0.002*	0.40	12.439	0.319	3.67
HIV stigmatizing attitudes						
Yes	1.000			1.000		
No	2.529	*0.001*	0.75	8.153	0.328	− 2.41
Constant				494.080	*0.000*	145.84
Total	0.194	*0.000*	57.211	0.145	*0.000*	42.79

Italic values signifies *p* < 0.05

According to the results in Table 3, differences in HIV Testing and Receipt of results between adolescent and non-adolescent women were significantly attributed to both differences in the characteristics and variation effects of predictors of the two groups (P < 0.05). Overall, about 57.2% of the gap in HTR can be attributed to differences in characteristics while 42.8% of the gap in HTR can be attributed to differences in effects of predictors or coefficients between adolescent and non-adolescent women.

Overall, Variation in the characteristics of adolescent and non-adolescent women contributed about 57.2% to the overall difference in HIV testing and receipt of results between adolescent and non-adolescent women. Specifically, the differences were significantly attributed to characteristics of women namely; education, place of residence, region, ever given birth, ever had sex, comprehensive knowledge and HIV stigmatizing attitudes (P < 0.05). The variation in these characteristics of women contribute about − 6.5%, 0.2%, − 0.5, 31.6%, 34.7%, 0.4% and 0.8% to the overall gap in HIV testing difference in adolescent and non-adolescent women respectively. The positive percentages in the results show the proportion in which the overall gap would reduce if the differences in the characteristics of women in the two groups were to disappear. On the other hand, the negative percentage shows the proportion to which the gap in HIV testing would increase if the differences in the characteristics of women in the two groups were to disappear. The overall gap in HIV testing and receipt of results would reduce by 31.6%, 34.7%, 0.4%, 0.8% and 0.2% if adolescent and non-adolescent women had similar proportion of their population in groups who have; ever given birth, ever had sex, comprehensive knowledge on HIV, HIV stigmatizing attitudes and place of residence respectively.

On the other hand, variation in the effects of characteristics (coefficients) contributed about 42.8% to the overall difference in HIV testing between adolescent and non-adolescent women. Specifically, the differences were significantly attributed to variation in the effects of characteristics of women namely; education and marital status (p < 0.05). The variation in the effects of characteristics (coefficients) of women contribute about − 68.6% and − 12.6%, to the overall gap in HIV testing change in adolescent and non-adolescent women respectively. In particular, the overall gap in HIV testing between adolescent and non-adolescent women would increase by 68.6% and 12.6% in the absence of variation in the effects of women’s education and marital status respectively on HIV testing. 57.2% of the gap in the rates can be attributed to differences in the constants of the models for adolescent and non-adolescent women. This suggest that the study variables may be unable to fully explain the gaps in HIV testing and receipt of results as some behavioral and cultural factors may be difficult to measure due to the nature of the data.

## Discussion

This study analyzed data from 2011 UDHS to assess differences in HIV testing and receipt of results between adolescent and non-adolescent women in Uganda. The findings show that differences in HIV testing and receipt of result can be attributed to both variation in characteristics (endowments) and variation in effects of characteristics (coefficients). In particular, the compositional variation in adolescent and non-adolescent women are noted in characteristics namely ever had sex, ever given birth, comprehensive knowledge on HIV/AIDS, stigmatizing attitudes, level of education and place of residence. Variation in the effect of characteristics (coefficient) also contributed to the changes for example variation in level of education and marital status though in a negative direction significantly contributed to differences in HIV testing and receipt of results between adolescent and non-adolescent women in Uganda.

The characteristics that had major contribution to the overall gap in HIV testing and receipt of results between adolescent and non-adolescent women were ever had sex and ever given birth. Having had sex explains the largest portion of the overall gap in HTR between the two groups. This points out to the issue of variation in perception of risk to HIV/AIDS between adolescent and non-adolescent. Adolescent women tend to perceive themselves as not being at high risk due to few exposure to hero-sexual intercourse which causes most of them not seek HIV testing. However this perception put adolescent women even at high risk of acquiring HIV in future as they undermine other sources of infections such as Mother-to-Child Transmission and sharing of sharp instruments. Besides, HIV testing has been found to be beneficial in reduction of risky sexual behaviors such as non-condom use, multiple sexual partnering, cross-generational sex, commercial sex and drug use among young persons. These benefits of HIV testing may be missed by adolescent women due to low HIV testing and receipt of results. A study conducted among young people in Nigeria reported that young people who have ever had sex were about 2 times more likely to go for HIV test than their counterpart who had never had sex [3]. This was still confirmed by a study conducted among young women in Tanzania which found that women who have ever had sex were 4.4 times more likely to test for HIV compared to those who have never had sex [8]. Besides, studies have indicated that HIV testing besides helping individuals to know their HIV statuses, serves to reduce unsafe behaviors [15]. This therefore point to the need to scale up HIV testing among adolescent women by increasing on awareness that focuses on clearing misconceptions about HIV among adolescent women.

Related to the above, having given birth was also found to be contributing a large portion to the overall gap in HIV testing and receipt of results between adolescent and non-adolescent women. This can be justified by the fact that majority of non-adolescent women have ever given birth and it is mandatory for couples to test for HIV during antenatal care in Uganda following mass campaigns on Elimination of Mother-to-Child Transmission of HIV (EMTCT). Less than quarter of adolescent women has ever given birth hence reducing the chances of testing for HIV compared to non-adolescent women. This reveals to government and development partners to scale up interventions targeting adolescent women for example in-school HIV testing counseling and testing and other adolescent friendly services.

Change in HIV testing and receipt of results between adolescent and non-adolescent women was also attributed to place of residence indicating that gap in HIV testing would reduce by at least 0.2% if this variation is removed. This can partly be attributed to concentration of health facilities in urban centers. This findings is consistent with a study conducted among young women in Tanzania which found out that young women with urban residence were 0.5 times more likely to test and receive their HIV result compared to the rural counterparts [3]. This finding directs government and other development partners to strengthen health services in rural areas in order to reduce the gap in HIV testing between adolescent and non-adolescent women in Uganda. However, this finding was contrary to studies conducted in Zimbabwe which did not find any significant relationship between place of residence and HIV testing [14].

Variation in level of education was also found in this study to have contributed to the overall gap in HIV testing and receipt of results between adolescent and non-adolescent women which is consistent with findings from a study conducted in Nigeria which revealed that young people with at least a secondary education are about 1.6 times more likely to access HCT than their counterpart with primary education or no education at all [3]. This finding was also confirmed from a study conducted in Tanzania which found that young women with secondary level of education were 5.5 times more likely to test and received their HIV result compared to those with no education [8].

## Conclusion

The findings of this study provide evidences about differences in HIV testing and receipt of results between adolescent and non-adolescent women. Government and development partners should scale up interventions aimed at increasing uptake of HIV testing among adolescents. Such interventions may include increasing community outreach services and establishment of adolescent friendly HIV testing and counseling centers.

## Abbreviations

AIDS: acquired immune-deficiency syndrome
ART: anti-retroviral therapy
HCT: HIV Counseling and Testing
HIV: human immune virus
HTR: HIV testing and receipt of results
MoH: Ministry of Health
PMTCT: prevention of mother to child transmission
UBOS: Uganda Bureau of Statistics
UDHS: Uganda Demographic Health Survey
UNAIDS: Joint United Nation Program on HIV/AIDS
UNFPA: United Nation Fund for Population Activities
VCT: voluntary counseling and testing
WHO: World Health Organization
